# How can robots facilitate physical, cognitive, and social engagement in skilled nursing facilities?

**DOI:** 10.3389/fragi.2024.1463460

**Published:** 2024-11-12

**Authors:** Rhian C. Preston, Madison R. Shippy, Carolyn M. Aldwin, Naomi T. Fitter

**Affiliations:** ^1^ Collaborative Robotics and Intelligent Systems Institute (CoRIS), Oregon State University, Corvallis, OR, United States; ^2^ School of Human Development and Family Sciences, Oregon State University, Corvallis, OR, United States

**Keywords:** socially assistive robotics, skilled nursing facilities, human-robot interaction, user-centered design, design thinking process, robots for older adults

## Abstract

As people live longer, the population of older adults in need of support continues to expand relative to the available workforce of caregivers, necessitating new solutions to supplement caregiver availability for the physical, cognitive, and social needs of older adults. Robotics and automation present strong possible solutions. Past solutions have typically supported short-term rehabilitation and aging in place, yet many older adults live in skilled nursing facilities (SNFs), a setting reached by relatively little research to date. In this paper, we examine the unique needs of staff and residents at SNFs, after which we begin an iterative design process of robot-mediated wellness activities for the SNF space. We worked closely with domain experts in exercise science and physical therapy for older adults and a local SNF to design and test a series of robot-mediated activity prototypes with residents, visitors, and staff. We found that while both residents and staff highly value physical activity, there are nuanced challenges associated with supporting resident activity (one important element of overall wellbeing). As a result, we considered and tested a wide range of intervention options from usual approaches (e.g., mirroring movements) to creative approaches (e.g., social engagement via lewd humor). Our final design insights can inform practitioners who wish to use robots to support resident wellbeing in SNFs.

## 1 Introduction

Due to the growing population of older adults, and ongoing difficulties with both hiring and retaining caregivers, the workload of caregivers in skilled nursing facilities (SNFs) has already exceeded a sustainable threshold and continues to increase (Hegney et al., 2019). SNFs are long-term care facilities for physically or cognitively impaired adults, typically in late life, who require 24-h nursing care. This high workload impacts time for training and onboarding, time spent with residents, and the overall ability to support resident needs and track resident health. Robotic assistance has long been an appealing prospect within various sectors of the medical field, and could be a useful tool for mediating health and wellness activities, with the potential benefit of mitigating staff workload demands as well as enabling resident-directed engagement.

**FIGURE 1:**
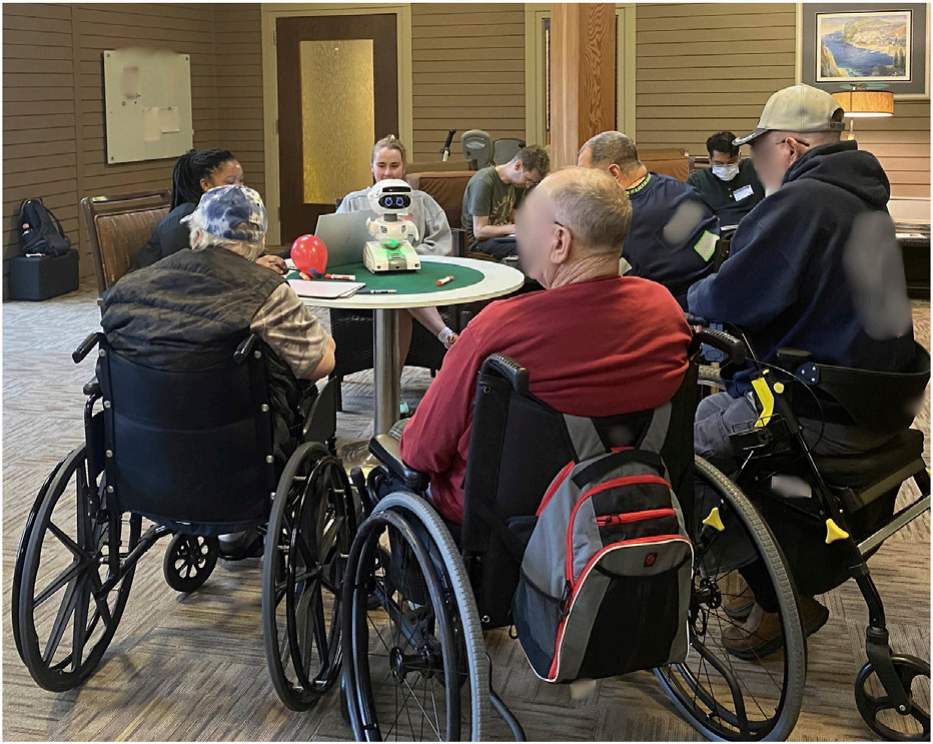
Residents interacting with one of the robot-based activities utilizing the Misty II robot.

Existing health-directed robotics work for older adults has often mirrored more clinical intervention methods in physical and cognitive therapy, with specific goal-oriented, structured interactions such as rehabilitation (Eizicovits et al., 2018; Feingold-Polak and Levy-Tzedek, 2021; Kashi et al., 2017; Kashi and Levy-Tzedek, 2018) or specialized memory care (Pino et al., 2020). Social interventions for elders have prioritized “companion” systems (Chang and Šabanović, 2015) and, more recently, telepresence systems (Cesta et al., 2016). Non-rehabilitation-focused robotics work such as gameified physical and cognitive exercise routines (Simão and Bernardino, 2017), and conversational partners (Pou-Prom et al., 2020) have been primarily tested with physically healthy and independent older adults who are often living on their own or with minimal assistance. These healthier older adults often have significantly different needs and capabilities compared to older adults in SNFs, such as better mobility or cognitive processing. As such, the majority of existing work on socially assistive robots for older adults expects individuals to still possess the same set of capabilities as a younger adult, but at a somewhat degraded capacity. To better understand factors unique to this population of older adults, we performed an iterative user-informed design process in collaboration with both experts in the field and the older adults *within SNFs* using a variety of robot platforms, such as the Misty II present in Figure 1.

Little robotics research to-date has explored the needs and perceptions of older adults in skilled nursing facilities, despite recognition of how perceptions towards robots differ across cultural backgrounds (Liu et al., 2021) and age groups (Raghunath et al., 2020). Of this existing work with more frail older adults, the majority has focused on specifically clinical needs, such as post-stroke rehabilitation (Langer et al., 2019; Feingold-Polak et al., 2021), or emotional and cognitive aids for individuals with advanced cognitive decline such as dementia (Pou-Prom et al., 2020; Hebesberger et al., 2016; Chang and Šabanović, 2015). To complicate matters, SNFs and memory care facilities, while predominantly supporting older adults, *are not exclusive to older adults*, and also include individuals who need similar levels of physical or cognitive assistance due to chronic illness or accidents. SNF residents are more likely to use wheelchairs, or have other types of limb impairments. Additionally, residents of SNFs often have more pronounced sensory and cognitive impairments (Yamada et al., 2014), and include significant populations of blind, deaf, or nonverbal individuals. Our robot-mediated activities, while similar to those in existing work with older adults and post-stroke rehabilitation, focus on how robots might support the needs of SNF residents without requiring staff initiation or facilitation.

In the presented work, we prioritize defining and better understanding the existing structures and challenges to supporting older adults in SNFs to meet their overall health and wellness goals, as well as supporting their autonomy and agency around their wellbeing. We followed a design thinking process that followed cycles of divergent and convergent phases to gather information from SNF stakeholders and iteratively prototype and test different robot activities to support residents of these facilities. This approach allowed us to cycle between eliciting challenges and needs of older adults/care providers, and exploring how these groups engaged with different robotic systems and activities. We present foundational information on SNFs, along with related work within assistive robotics for older adults in Section 2. We provide information about our partner facility and frame our collaborative design process in Section 3, before presenting the divergent data-gathering steps and results in Section 4. Section 5 provides information on the prototype generation and selection process, and Section 6 presents the prototypes and testing results. Lastly, we synthesize the results of the design thinking process, present a set of needs and associated challenges for SNFs, and share guidelines for roboticists interested in the SNF application space in Section 7. This description of existing needs and challenges, as well as guidelines for designing and implementing robotic systems within SNFs, are key contributions of this work. While several of the presented guidelines are generally known and documented in other research communities, they have either not seen broad adoption within the robotics community, or require further consideration for the SNF domain specifically.

## 2 Related work

To appropriately frame this work, we must first explain what is unique about skilled nursing facilities in addition to examining past robots and support systems for older adult health and wellness.

### 2.1 From aging in place to skilled nursing facilities

Aging in place has been a key area of research both within healthcare and ancillary fields such as robotics, however, aging in place relies heavily on having a support network of friends and family. Additionally, late life can extend over several decades, and the needs of individuals will change as they age, so aging in place does not necessarily remain feasible for all of their life. When people are no longer able to remain independent, either from age or from other medical complications, skilled nursing facilities (SNFs) typically become necessary within the cultural context of the U.S.

There are over 15,000 SNFs in the U.S. alone (Harrington et al., 2015), with small facilities having 50 or fewer beds, and large facilities having over 150 beds. SNFs have onsite Certified Nursing Assistants (CNAs) and registered nurses (RNs) to monitor the health and wellbeing of residents (Yee-Melichar et al., 2014). These residents require a high level of care and attention due to severe health declines, chronic illnesses, or other health-related events. Generally, individuals in SNFs require assistance with two or more activities of daily living, such as feeding and bathing. SNFS can also include individuals that have been released from hospital care, but still need much more attention and support than they could receive either at home or another facility (e.g., rehabilitation centers) (Yee-Melichar et al., 2014). SNFs manage and track all aspects of a resident’s wellbeing, from medication intake to social engagement (Pennington et al., 2003). These tasks can be time intensive; for example, transfers, in which a resident needs to be moved from bed to wheelchair or wheelchair to shower, can often require multiple CNAs to perform (Beer et al., 2011). CNAs also track social activities, such as checking with each resident on their desire to participate in events and overall mood (Dobbs et al., 2014). Additionally, CNAs lead functional maintenance activities, such as assigned physical therapy (PT) exercises intended to preserve and extend resident capability. By delineating this information, we intend to highlight the unique challenges to resident capability, resident agency, and CNAs’ task overload within the SNF context. In consideration of these factors, any robotic system that we introduce to an SNF should have minimal barriers to resident engagement, and should at a minimum not require staff support for usage.

### 2.2 Wellness robots

Past studies have sought to support physical, cognitive, and social wellness needs for older adults. For example, past assistive robotic research has explored areas such as post-stroke rehabilitation (Langer et al., 2019; Feingold-Polak et al., 2021; Eizicovits et al., 2018), walking companions (Hebesberger et al., 2016), reminder systems (McColl and Nejat, 2013), and exercise or dance coaches (Fasola and Matarić, 2013; Caic et al., 2019). Here, we focus specifically on systems that have been studied within SNFs or domains relevant to those within SNFs, such as post-stroke rehabilitation.

#### 2.2.1 Physical wellness

Work in post-stroke rehabilitation has highlighted concepts such as mirroring, where participants match the movements of the robot, as an additional clinical exercise to improve limb function (Feingold-Polak et al., 2021; Kashi et al., 2017), but mirroring activities have also been used to promote social grouping and feelings of attachment within HRI generally (Feniger-Schaal et al., 2018). For general older adult populations, mirroring has been used with robotic exercise instructors as a game, as well as for teaching exercise poses (Fasola and Mataric, 2010). For individuals with dementia, mirroring has been used to teach dance moves (Schrum et al., 2019).

#### 2.2.2 Cognitive wellness

Existing cognitive support robots include robots that play mental games (e.g., tic-tac-toe or chess). While these games could feasibly be played with a virtual opponent or on a computer, existing work has found participants prefer systems that physically interact, rather than screens or tablets, and these physical systems can also prime participants to respond (Eizicovits et al., 2018). Past work has also examined learning activities as a method to support cognitive function (e.g., foreign language training) (Wong et al., 2019).

#### 2.2.3 Social wellness

Past works have explored how people perceive robots as social agents in assistive roles such as exercise coaches (Čaić et al., 2020), or ways for a robot to be perceived as more social (Noguchi et al., 2022; 2018). However, a great deal of work has also directly examined robots for social wellness, primarily as companions such as robot pets or through one-on-one interactions, but also through enabling interaction between people (Gasteiger et al., 2021; Thunberg, 2024). Robot pets have been extensively explored, especially for use with dementia patients (Abbott et al., 2019; Bradwell H. et al., 2021; Chang and Šabanović, 2015; Hung et al., 2019), and have been primarily cited as useful interventions for agitated or distressed individuals. General design recommendations for social robots have also been explored, such as improved robot mobility, voice recognition, ease of use, and soft, friendly aesthetics, though such work has primarily centered on healthcare professionals (Bradwell H. L. et al., 2021).

Older adults themselves, despite suffering from high levels of isolation (Nicholson, 2012; Shankar et al., 2011) and feelings of social disconnection (Cornwell and Waite, 2009), will also utilize whatever tools are available to build social support structures and connect with others (Richards et al., 2021). This observation has led to examining robots as social facilitators, (e.g., to help handle tasks such as social scheduling, transportation, or game facilitation) (Ling et al., 2022). Telepresence robots, which allow individuals to videoconference with and navigate a distant space, have been the primary method to explore social facilitation, but more recent work has also begun examining and defining how robots could act as local social mediators (Chita-Tegmark and Scheutz, 2021), which is particularly useful for individuals in SNFs.

Approach in our work: These existing works frame the challenges of designing robots to encourage or support healthy physical, cognitive, and social wellbeing, and present a wide variety of existing assistive robots and activities. We utilized this existing body of work on developing games and activities to support older adult wellness and rehabilitation, layered with the insights and methods that explore enabling social interaction between individuals, as a basis that guided our initial investigation approach and activity concepts. Compared to these past works, we sought to advance usability and potential for end user autonomy in our design process.

## 3 Partnering facility and design process

For this work, we partnered with a local SNF to better define the unique needs of the facility and their residents, and develop and test various prototype systems to support them. Our partner facility is larger than average, with ≈150 beds compared to the average ≈108 beds (Harrington et al., 2015), and specifically supports military veterans. The residents are all veterans or spouses of veterans; as such, the population is predominantly male (≈80%) . The average resident age is 79, with over 80% over residents over the age of 70. This particular resident population is predominantly wheelchair users and individuals with various types of limb impairments, and a subsection of the population is nonverbal or deaf. Additionally, over half of the residents have some form of dementia, and the facility has a dedicated memory care unit for those residents with more advanced stages of dementia.

While working with our partnering facility to identify needs and possible support system solutions, we chose to follow an iterative design method based on the design thinking process (Thoring and Müller, 2011). This process has broad applicability and focuses heavily on user-centered and user-collaborative design principles, which aligns with recommendations to more deeply involve older adult residents with the overarching design process (Frennert and Östlund, 2014; Bardaro et al., 2022). Past design thinking-based assistive robotics work includes the development of Vizzy, with its augmented reality exercise games (Moreno et al., 2016; Simão and Bernardino, 2017), and Stevie, a socially assistive robot for retirement communities (McGinn et al., 2020). Design thinking focuses on divergent and convergent steps, usually centered on five main types of effort: empathize, define, ideate, prototype, and test. The divergent phase includes the “empathize” step, which focuses on researching user needs, and the “define” step, which involves digesting user need information to identify clear problems at each iteration. The “ideate” phase is often a bridge of sorts between the divergent and convergent phases; this step involves challenging assumptions and creating ideas. The convergent phase includes the “prototype” step, which involves building solutions in a range of fidelity levels, and the “test” step, which is actual testing of the prospective solutions. The full process as implemented in this work is presented in Table 1, which at a high level describes the goals at each step, the types of activities we performed as part of that step, the methods we used, and the associated outcomes.

**TABLE 1:** Design process of the robot-based activities following the design thinking model, including the goal, type of activity, methods used, and high-level outcomes of each design step.

	Empathize	Define	Ideate	Prototype	Test
Goal	Elicit information about health needs of older adults and unique characteristics of (and challenges in) SNFs	Identify broad needs and requirements for robotic systems and activities meant to support older adult health in SNFs	Generate system and activity ideas	Rapidly develop system and activity prototypes based on the ideate phase	Evaluate how older adults in SNFs and staff react to and engage with prototype systems
Activity Type	Conducting observations and interviews	Synthesizing empathize results and developing system design requirements	Brainstorming with research team and collaborators	Developing hardware, software, and interactions	Facilitating group sessions with prototype systems
Methods	Note-taking on behavioral observations and interviews with experts	Note sorting, consolidation, and supplementation	Generating broad system and activity concepts	Developing hardware and software	Collecting, sorting, and consolidating field notes, participant comments, and staff feedback
Outcome	Understanding of experiences and unique characteristics of SNFs and their residents	System and activity design requirements	List of candidate robotic platforms and candidates for activity prototypes	Six robot-mediated intervention prototypes motivated by previous steps	Synthesized design insights for use by others working in related research areas

## 4 Divergent design steps: Empathize and define

Our divergent phase included two separate empathize steps: 1) observation of activities within the SNF and 2) interviews with SNF wellness-relevant experts, whose methods are presented in Section 4.1 and Section 4.2, respectively. The results of the activity observations and expert interviews are then presented as our major define step in Section 4.3, and the summarization of key highlights of the define step are presented in Section 4.4. Our university ethics board approved these activities.

### 4.1 Empathize: Social activity observation methods

As part of our initial examination, we performed fly-on-the-wall and participatory observations of two staple social events (poker and bingo) at our partner facility. Both events are social events that include both cognitive engagement and fine motor skills-based manipulation. Poker was selected based on staff statements that the poker events in particular become very animated, with a great deal of banter. The bingo events were selected for having a much larger resident turnout. While they are less inherently social, they were nevertheless described as a very important activity for the residents broadly.

Members of our team observed three poker events and one bingo event to better understand how residents interacted with one another in these social settings. The poker events were conducted in the morning, at a table in an open common space with multiple entrances within the facility. For one event, one member of the study team attended as an observer. For the other two poker events, one member of the study team attended as a player. Bingo was conducted in the evening in the main larger activity hall, and two members of the study team attended as observers over the course of 2 hours (four games).

During the observations, the study team members detailed the demographics, level of interaction (how many people were interacting), types of interaction (cooperative, antagonistic), and language used (antagonistic, encouraging). We recorded data as handwritten notes, with initial notes during the events that were expanded into more detailed notes after the event.

### 4.2 Empathize: expert interview methods

To better understand the current difficulties within SNFs, and how to support older adult wellbeing, it was important to speak with a variety of experts. We spoke to experts who focused on a range of topics, including general gerontology, social support services, exercise science, and PT. Our expert interview participants were recruited via snowball sampling, as supported by our gerontology collaborators and partner SNF.

**FIGURE 2:**
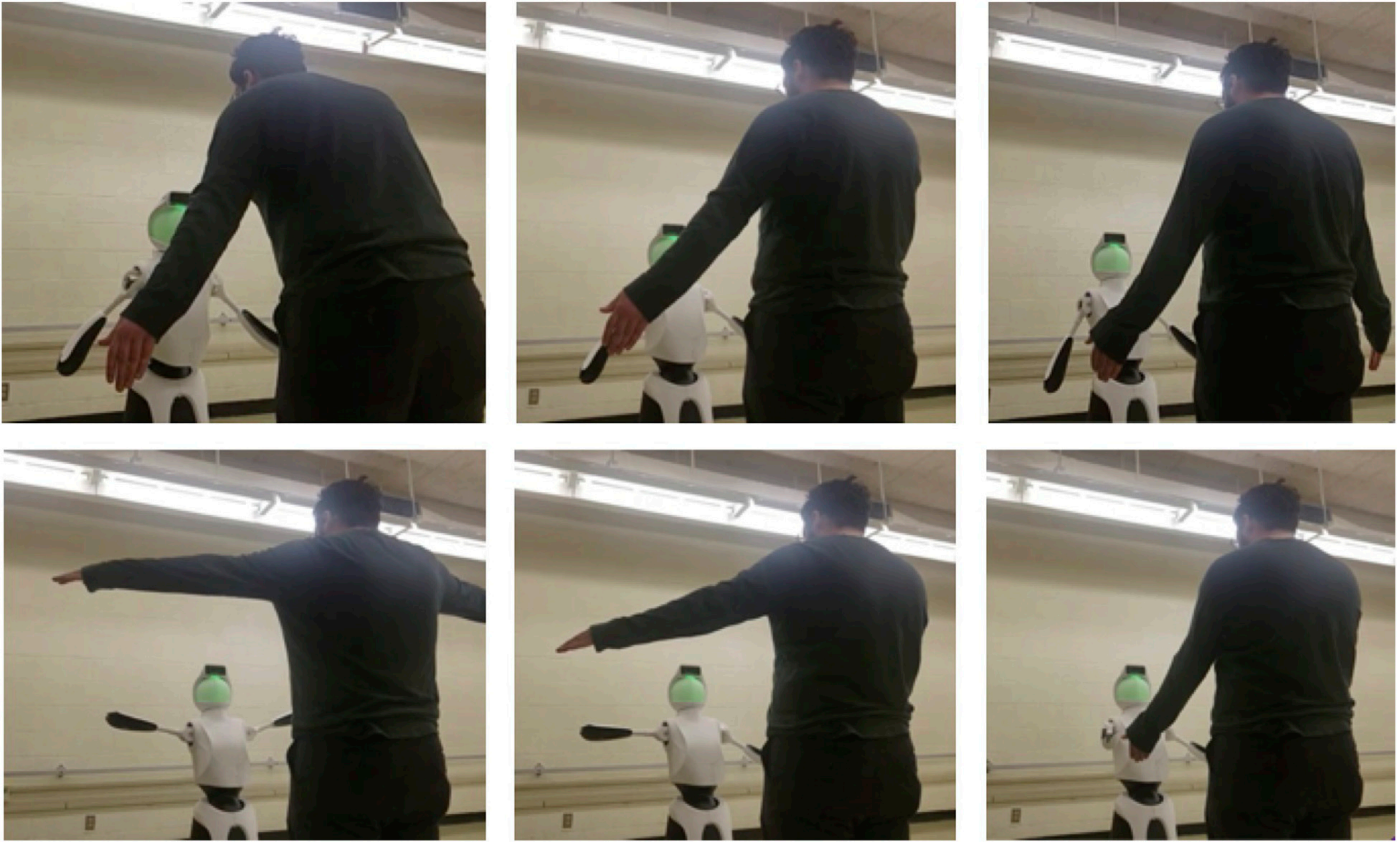
Images of key movements in the Quori demonstration video.

Our four initial hour-long interviews, as facilitated by a team including roboticists and gerontologists either through Zoom or in person, focused on physical health and exercise needs. We spoke with three physical therapists, including the director of rehabilitation at our partner SNF, as well as an exercise scientist specializing in older adult care and healthy aging. We asked semi-structured questions about general health needs and concerns, including differences between rehabilitation and health-maintenance-focused PT, barriers to adherence of exercises, and how robots could be used to support physical health. We presented video of a Quori robot (as further explained in Section 5 and shown in Figure 2) exercising with a mock user as part of the discussion of robot assistance. As part of one PT interview, we also requested a mock demonstration of PT sessions as typically performed at our partner SNF, including the PT exercises used for health maintenance.

An outcome of these initial conversations included more understanding of the interplay of physical health with other aspects of overall wellbeing, which led us to broaden the set of interviewed experts to include three additional participants: two members of the social support services team and the head of the recreation and activities team at our partner SNF. These additional semi-structured conversations focused on social activity levels, mood, and overall wellbeing of residents, as well as appropriate types of activities, to help us understand associated needs, existing limitations, and how to generate engagement.

We recorded all interviews through handwritten notes, with initial notes during the conversations that were expanded into more detailed notes supplemented by working documents and related literature after the interview.

### 4.3 Define: Results from empathize and data consolidation

We consolidated the data collected during and after the observation sessions and expert interviews, iteratively organized this information into broad themes, and confirmed the themes and their content with our experts. From the three poker sessions and one bingo session, we identified interactions between participants and noted whether these interactions were between residents and residents, residents and staff, or staff and staff. We also categorized the type of interaction as providing assistance, teasing, or general conversation. For the expert interviews, we grouped their responses by overarching theme. The themes that arose included items for quality of life considerations, resident autonomy, barriers/enablers to resident engagement, and the role of socialization. A second trained coder reviewed approximately 20% of the data to confirm inter-rater reliability; the Cohen’s Kappa value was 0.89, which indicates near perfect agreement. We first present the results of the social activity observations, and then the results of the expert interviews. The interview results and themes in particular became quite nuanced, and are presented primarily through distinct consolidated take-away points. The results of this define step highlight the needs and existing difficulties that impact the health and wellbeing of older adults in SNFs.

#### 4.3.1 Social activity observations

The first poker event had six players (5 male, one female), and an older male military veteran non-resident as the volunteer poker dealer. The volunteer dealer in the poker game tried to keep the residents engaged by telling stories and jokes. We observed minor teasing commentary from the other players and dealers towards one player who had the most chips, and they repeated these comments for the player when he could not hear them. We observed cooperative behaviors during the poker game, such as residents with more dexterity helping others to adjust card placement and bet with the correct chips.

The second poker event had three players (2 female and one male), and the third had four players (all male). For both of these events, a female staff member of the SNF was the poker dealer. The staff dealer in the poker games tried to keep the residents engaged by telling stories and jokes. Occasionally, the dealer would tell an uncouth joke, which yielded laughter from all the residents. The female residents in the second event were more social towards the staff dealer than the residents in the third event. We observed only a few non-game-related interactions between residents over the hour-long poker games; the residents interacted to help progress the game, but most banter was directed towards the dealer. Additionally, when another staff member came to talk with the dealer for a while, players did not engage in parallel conversations.

The bingo event was much larger, with around twenty participants in attendance and a roughly even split of male and female participants. Only two participants were not in wheelchairs. Participants were seated at tables in a U-shape facing the bingo caller, and each participant had either four or six bingo cards in front of them. The bingo caller was the same female SNF staff member from later the poker games, and generally there was about 10 seconds between number calls. The caller tried to engage participants by telling inside jokes and stories with the residents, but there was little reciprocated interaction. Residents did, however, form small groups of two or three based on where they were sitting. Within these small groups, players spoke to each other and helped fill out each other’s bingo cards throughout the game. One player won four games in a row, which we expected to generate some reaction from the other players in their the group, but that did not happen. Participants did not rotate groups during the bingo events.

#### 4.3.2 Expert interviews

The information highlighted by our experts varied based on their particular domain of expertise, as expected. We consolidated their insights by overarching associated take-away points, as presented below.

Our initial conversations involved primarily physical therapists, who provided nuanced information on barriers to adoption and adherence of exercise routines, ways to mitigate these barriers, and pointed information on key types of exercises that are important for supporting autonomy of more physically fragile older adults. Additionally, our exercise science expert, and follow-on conversations with social support services and activities staff, strongly highlighted the social needs of the residents, and the overall impact of social interaction on general health and wellbeing (“being social is a health protectorant.”) Lastly, when shown the Quori demo interaction video and asked about uses for technology in particular, the experts provided several pointed comments related to both designing activities, as well as the form and interaction modes these systems should exhibit.

Ultimately, these iterative interviews and the information provided by the seven care providers and experts were consolidated into six categories of take-away messages.

Take-away 1: The barriers to getting SNF residents to be physically active are multi-faceted and often independent from desire. The experts articulated a multitude of barriers to both starting exercise, as well as adhering to exercise and PT maintenance regimens. Some of these barriers were well known, such as people seeing exercise as repetitive or boring, or limited short-term outcomes inhibiting motivation (Lees et al., 2005). Ongoing pain, fear of injury or falling, and high levels of depression associated with isolation, are also common barriers, especially in care facilities (Cohen-Mansfield et al., 2003). Other barriers were more nuanced, such as the very term “exercise” raising assumptions about the difficulty, enjoyment, and required capabilities associated with an activity. This term also elicits concerns about being judged or “good enough” to start, which causes individuals to remain detached from the long-term benefits and to avoid starting altogether.

A notable number of mentioned barriers were closely linked to health limitations and SNFs. CNAs, who each have a specific case load of residents, are required to facilitate and track completion or refusal of all routine PT exercises assigned to each resident. This responsibility is on top of all other tracking and support tasks, such as logging biometrics, meals and food intake, sleeping time, and restroom usage. This high workload results in increased turnover in staffing (Castle and Engberg, 2006; Kennedy et al., 2020), which leads to inconsistency in both the training CNAs receive, as well as the degree of prioritization of PT tasks across CNA shifts throughout the day and week. Residents of SNFs also often have significantly reduced mobility and thus have an increased need for physical assistance, which can limit residents’ abilities to perform activities on their own. This reduced mobility and functionality, paired with the hands-on CNA roles, can generate feelings of helplessness that prevent residents from exercising agency. Additionally, residents with cognitive decline may not remember the individual exercises, nor remember the benefits or purpose of the exercises, needing to be reminded of both. Cognitive decline also results in declining ability to sustain attention, which leads to additional difficulty keeping the individual engaged or focused on the activity.

Take-away 2: Enabling physical activity relies on empowering residents and minimizing additional workload on staff. While discussing these barriers to exercising, the experts we spoke with also provided methods to enable exercise that are known within their fields, as well as a variety of existing processes to try to overcome exercise barriers. One key method mentioned was social enablers. Social enablers were best described by our exercise science expert as the idea of having a friend or close acquaintance encourage the individual and exercise with them. This process provides both a social safety net against the concerns associated with exercising, as well as acting as a distraction against the possibly repetitive or boring nature of an exercise, which makes the whole experience more enjoyable. The exercise scientist emphasized the value of a peer relationship, when the more experienced or invested partner would, at least at the beginning, be the instigator to lower barriers to entry.

Group exercise participation was one method that was discussed for encouraging physical activity. Our experts noted that group therapy and activities in nursing homes are used to improve self-efficacy of the participants. Additionally, group activities allow participants the option to watch the activity and build up to engaging. A round-robin style of leadership or a “peer-only” activity can further improve the overall engagement of participants. Group therapy, and socialization itself, also acts as a dissociative tactic to distract from the physical exertion. Comments on the value of group therapy were tempered with the addendum that group exercise participation is impacted by the mannerisms of the instructor and the culture of other participants in the group and that the attention of one-on-one interactions can give people a sense of safety if they lack confidence in their ability.

Our exercise science expert in particular identified the value of social interactions as a “health protectorant” and discussed the value of social activities in maintaining health. They provided examples such as “knitting or storytelling groups” that include built-in social elements that could well be incorporated into traditional PT and clinical focuses. They also pointed out that engaging individuals in healthy activities and behaviors first requires addressing social needs, and that these individuals are more likely to be suffering from loneliness and a lack of physical interaction. Social support also provides methods to engage and enable individuals who are “moderately interested” in physical activity, as they often want to engage, but lack confidence or the necessary feelings of safety to do so.

Beyond the use of social interactions, there were several process methods that were utilized within SNFs. One particular process was minimizing the variations in exercises that the PT assigned for each of the older adult residents. This reduces the cognitive load on the CNA when leading residents through exercises, since the differences are number of repetitions or weight load, instead of the exercises themselves. Additionally, for residents with some cognitive decline, the staff members maintain a form which explains the purpose of the exercises and the specific benefits for an individual resident, in order to remind them when they forget while doing these activities. The staff also talks through each activity while it is performed to assist in maintaining attention.

**FIGURE 3:**
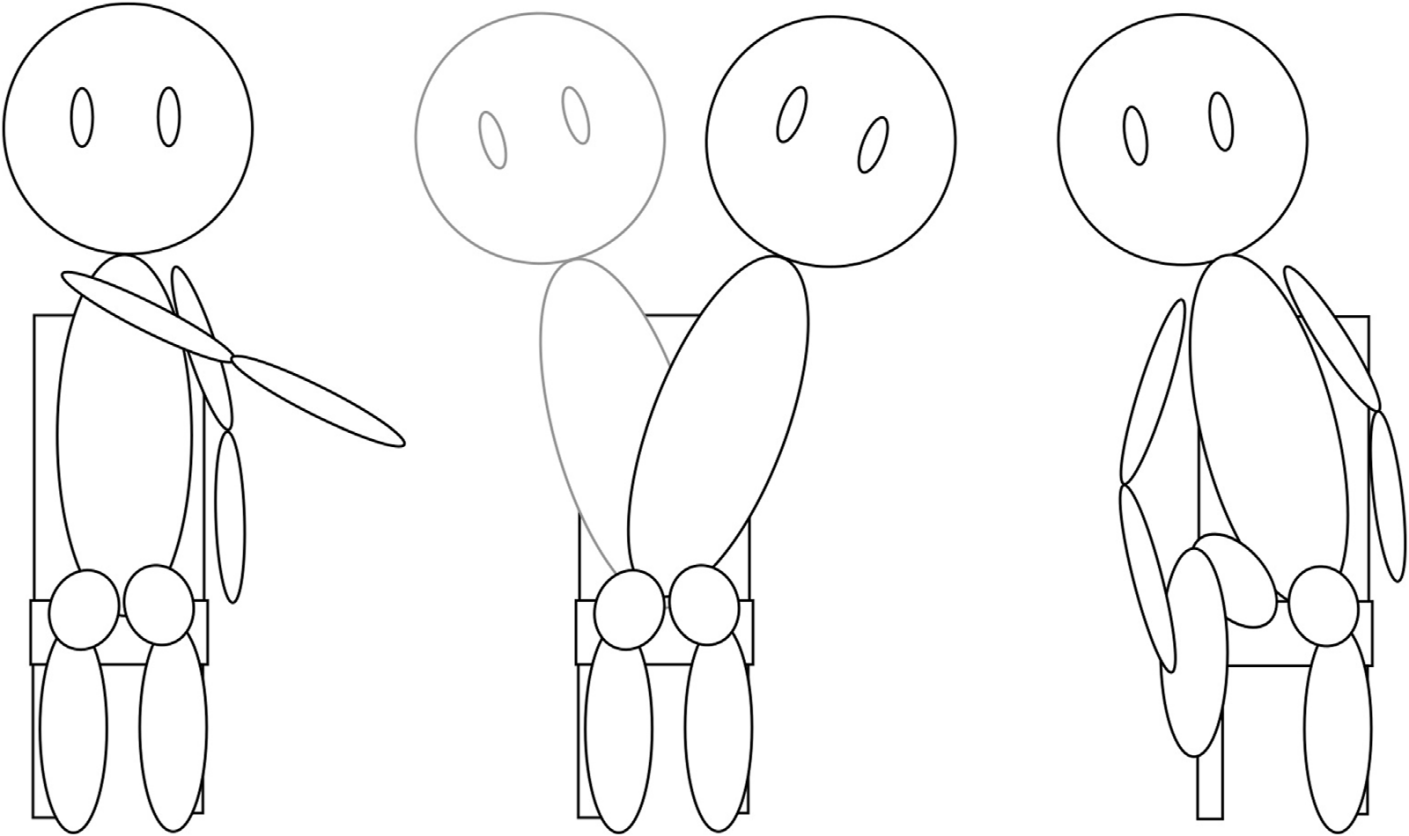
Representations of the three main exercise groups discussed by our experts. From left to right we have cross-body reaching, weight shifting/waist movements, and foot reaching/leg movements.

Take-away 3: There are key types of movement that should be prioritized to support resident autonomy. A key point discussed was the specific physical therapy exercises that are important maintenance routines to prolong key autonomy needs for older adults, especially those in SNFs. The information can be generally distilled into three key types of exercises: cross-body reaching, weight shifting/waist movement, and foot reaching/leg movements, which are described below and visually presented in Figure 3.

• *Cross-body reaching* is important to help maintain shoulder and elbow range of motion, as well as overall range of motion, and can limit the loss of function in the event of stroke or other single-side injury.

• *Weight shifting and waist movement* were highlighted as especially important for transfers, which is transitioning between sitting and standing positions or between chairs. The SNF physical therapist emphasized this idea, as improving or maintaining this strength can decrease the number of caregivers necessary for someone to complete a transfer, in turn decreasing the time to complete a transfer task, which improves care quality for the older adult and reduces the burden on caregivers.

• *Foot reaching and leg movement* exercises were the third mentioned area of focus. The ability to lift or pull up ones feet allows for putting on or taking off socks and shoes. Additionally leg and ankle strength exercises, even for older adults in wheel chairs, supports balancing in transfer tasks and self-ambulation.

Take-away 4: Meeting resident social needs has a direct impact on both their overall health and ability to engage in healthy behaviors. As noted previously, the exercise science expert considered social interactions a health protectorant, and the social support services experts and activities team lead strongly agreed. These individuals repeatedly mentioned the importance of getting residents out of their rooms to interact with others, and the frequent difficulties in doing so. They also brought up the day-to-day inconsistencies related to mood, energy, and sociability levels of individuals. They noted that residents have more energy in the mornings, and are also often more cognitively aware at that time, making it an ideal time for socialization and more energized activities. They also noted that many of the residents desire more conversation, or opportunities to share stories, and that even the non-verbal residents often enjoy joining groups to listen to the conversations and stories of others.

Take-away 5: Activities should be structured to build social comfort early on, and designed so participants can engage at a variety of levels. The experts we spoke with also provided specific ideas about types of activities and prospective assistive robots, especially after viewing the robot demonstration video. One aspect noted was that upper body work raises the heart rate more quickly than lower body workout tasks, which should be considered in activity length. Similarly, interaction sessions should start with a few-minute section that is robot lead to help participants with social comfort and understanding about the activity. If we use activities that participants might be dismissive of, such as meditation or yoga, we should consider using a different name for these activities to mitigate negative assumptions. One such activity discussed with experts was mirroring or pose-matching tasks, which are popular in both rehabilitation and team-building. Our experts noted that mirroring tasks are good for chronic pain, and that even watching the robot do the movements or imagining themselves doing the movements can improve how well individuals perform the action later. Benefits of multi-tasking can also be achieved this way, by having participants perform cognitive or imaging tasks while performing a physical activity. Another note was that tactile interaction can be a useful feedback mode, as well as enhancing engagement, introducing activity props such as throwing a balloon or ball or giving and receiving items can engage participants.

Take-away 6: The form and presentation of a robot and the activity must be visually understandable and approachable. The experts shared feedback related to the form the robots should take. Many notes were related to both visibility of the robot and legibility of the robot’s actions and intent. These notes included comments about larger robots being easier to see, concerns about screens or tablets not providing a clear representation of visual depth, and the value of the robot motion and degrees of freedom matching a person’s movement structure to better communicate intent. The experts also noted the value of “cuteness” in both drawing and holding participant attention. Residents of SNFs are more likely to have impaired mobility, and our experts discussed residents having misgivings about wheelchairs and the association with a loss of independence. The experts mentioned that seeing the robot use a wheelchair or exhibit degrees of freedom similar to wheelchair users could help normalize their own wheelchair use.

### 4.4 Key highlights

From the social activity observations, we noted trends in participant interactions with the activity and each other. Participants tended to take initiative when supporting each other, which aligns with existing work examining how older adults build supportive social communities among themselves with whatever tools and methods are at their disposal (Richards et al., 2021). We also noted participants tended to be somewhat more social in their responses to non-staff *versus* staff activity facilitators between the first poker session and later sessions. The results of the expert interviews reinforce this notion highlighting the importance of peer relationships in encouraging active engagement, and difficulties with learned helplessness in initiating engagement. Our define step showed that social interaction is crucial for both physical and cognitive wellbeing, as well as willingness to engage in healthy activities. The experts’ design guidelines can help us create systems appropriate for older populations with declines in visual and auditory acuity, as well as cognitive processing declines.

Through this process, we were directed to look at older adult wellbeing as a whole, and we used this idea to determine a set of broad requirements to guide our ideation and development phases. As these requirements are intended for overall wellbeing, they should include a mixture of physical, cognitive, and social components, as captured below.

• Require either a fine or a gross motor function task

• Require either a physical touch or a social interaction component

• Work in both individual and group settings

• Work for a broad range of cognitive and physical capabilities

• Drive participant engagement and re-engagement

These high-level requirements from the define task were then carried into ideation, where we began formulating robot activities.

## 5 Ideation

The results of our divergent process fed into the ideation step, where we began conceptualizing various activities and systems to implement and test at the SNF. These ideas were generated among the research team, and expanded or eliminated as we began the prototype phase and asked more critical questions about what should be tested at our partner SNF. Our research team member from gerontology played an active role during this conceptualization process, providing both new activities and activities already adopted by the field, as well as feedback on validity or specific requirements for activities generated by other members of the team. Based on define phase outcomes, our overall intervention goals were focused heavily on improving self-efficacy, decreasing feelings of helplessness, and encouraging more overall participant activity.

### 5.1 Robots

**FIGURE 4:**
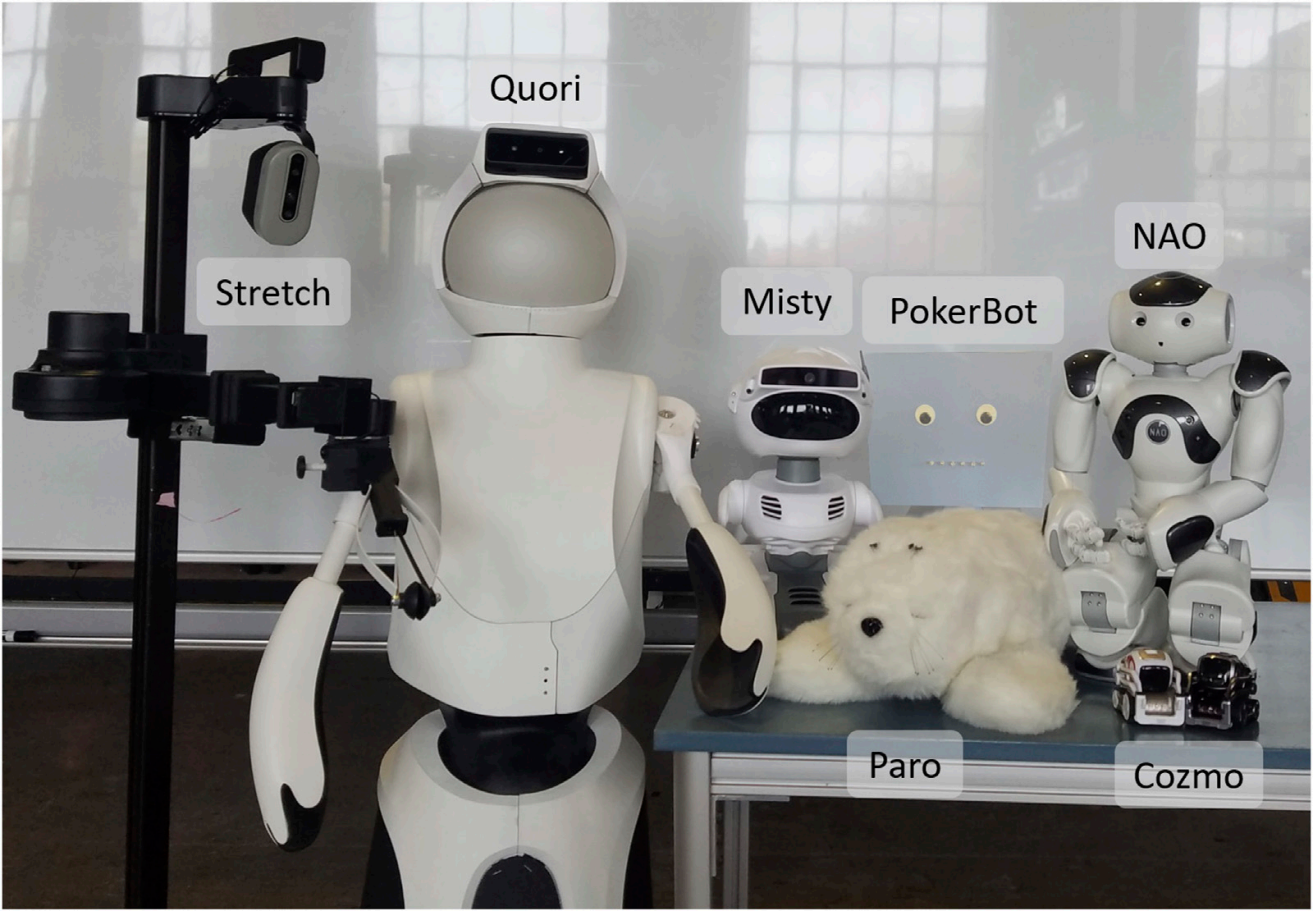
Images of the robots considered when developing activities. In the back row from left to right: Stretch, Quori, Misty, PokerBot, and NAO. In the front row are Paro and two Cozmos.

We considered a broad set of robotic platforms for use in prototyping activities. These systems included Paro, Stretch, Misty, Cozmo, PokerBot, NAO, and Quori, as visually presented in Figure 4. These systems were chosen based on the social design, such as Misty, Cozmo, and Quori; systems that have been commonly used within the SNF space, such as Paro and NAO; and capabilities that aligned with feedback from the define step use cases, such as PokerBot. Each considered system is further described below.

Paro is a robotic seal intended for touch-based emotional support interactions, and it is already commonly in use with older adults with cognitive decline. The robot itself has limited functional capabilities, and is not intended for modification, but it does have reactive behaviors that promote continued interaction with the robot, such as reactive motion and audio. We selected Paro because it is a well-researched system designed to support individuals with dementia, and is therefore well-suited to the SNF space (Hung et al., 2019). This type of system can offer a useful reference point for more exploratory options.

Stretch is a mobile manipulator with an extendable compliant gripper designed for in-home and assistive tasks. Stretch is non-social in design, but it has been used in physical-therapy-related research and assistive teleoperative tasks for individuals with low mobility. We selected Stretch based on its usage within in-home and in-care-facility assistive robotics research (Olatunji et al., 2024).

Cozmo is a very small social robot with a wheeled base, expressive screen face on an up-down pivot head, and a small front lift. Cozmo has been used in social and behavior change research, though this work has been primarily with children. We selected Cozmo due to its pet-like social qualities and usage in socially-assistive work (e.g., efforts focused on nudging (Preston et al., 2023) and social facilitation (Gillet et al., 2020)).

Misty is a small social robot with a large expressive screen face, a pose-able head, a mobile base, and two small arms that are intended to aid in expressive motion. We selected Misty because of its expressive head motion and well-defined conversational capabilities.

PokerBot is a custom robot for poker companionship with a bust-like form factor. It is designed to provide verbal information about the current state of the poker game, as well as to use verbal commentary to encourage social conversation among players (such as through banter and pun-based jokes). We selected PokerBot based on staff comments about the importance of poker play and resident enthusiasm for Poker as a social activity. Additional specific details about the design of this robot are presented within the prototyping step of the design process, in Section 6.6.

NAO is a small humanoid robot standing at about two feet tall, with a static face. NAO has been routinely used in both social and assistive robotics research across all age groups. It has seen particularly frequent use for exercise demonstrations due to its extensive range of human-like joint articulation compared to other commercial robots. We selected NAO based on its usage for socially assistive robotics research with older adults (Trainum et al., 2023), as well as its kinematic similarity to humans, which is ideal to support tasks such as PT demonstrations.

Quori is a full-sized humanoid robot, with a wheeled base and back-projected face, that was designed specifically for human-robot interaction research. Quori can bend forward and backward at the waist and has compliant shoulder pitch-roll joints, but it lacks elbow and wrist joints. We selected Quori due to its expressive facial characteristics. We also believed its larger form factor could make it easy to see.

In the ideate phase, we focused on the applicability of each platform with potential activities, as well as the limitations and expectations of both the SNF residents and facility.

### 5.2 Activities

To match our concrete requirements from the define step we devised a broad list of activity concepts for fine/gross motor control, social interaction, and cognitive engagement, based on both information collected during the previously discussed observations and interviews. We also used existing work from both gerontology and robotics to guide design choices and consider how existing past activities perform with less physically-healthy older adults. Potential activity concepts included touch-based therapy, directed and undirected conversations, card games, controller-based cooperative and competitive games, pose matching or mirroring, dance, and yoga. We paired these activity concepts to the available robot platforms to determine best-fit, as aligned with existing design process recommendations (Bardaro et al., 2022), such as the use of Paro for touch interactions, the host of social robots for conversations, simple mobile systems such as Stretch or Cozmo for teleoperated games, and NAO for the more human-motion-matched activities such as mirroring or dance. We then tailored the resulting combinations to lean into humor and playfulness, and downplayed scorekeeping or corrective feedback, to position the robot as a peer rather than a coach. The resulting prototype activities are explained in detail in the following section, followed by the results of the corresponding testing.

## 6 Convergent design steps: Prototype and test

Once we determined which conceptual activities to pursue, we began creating initial robot-based activities to test with the residents of our partner SNF. Six activities were implemented, using six different robotic systems. These prototype systems were then tested at the SNF, and all tests followed the methods described in Section 6.1. The descriptions of each prototype system and the corresponding anecdotal test observation results are described in Sections 6.2 through 6.7. Lastly, the synthesis of the key results from this process are presented in Section 6.8. All presented activities were approved by our university ethics board.

### 6.1 Methods for evaluating all activities

These activities took place over the course of 6 months through nine 1-h sessions open to all residents, staff, and visitors to the SNF. Some activities were incorporated multiple times, and others were only used for single sessions. We took hand-written notes on observations during these sessions, focusing on interactions between participants and engagement with each robot activity.

The number of participants at each event ranged from two to eleven. The majority of participants were male (46 men, eight women over all sessions), and used wheelchairs (43 wheelchair users over all sessions).

We examined the notes from these activities for broad themes, including highlighting similar/different trends across activities and any unique aspects of a given interaction. These broad themes centered on participant-participant interactions, participant-system interactions (including distinctions between observer and active participants, as well as re-engagement), and participant-participant-system interactions (e.g., when participants used the system as a method to engage with other participants). A second trained coder reviewed approximately 20% of the data to confirm inter-rater reliability; the Cohen’s Kappa value was 0.87, which indicates near perfect agreement. The results are presented along with each activity.

### 6.2 Group social interactions with Paro

Paro’s usage was predicated on the body of existing literature around the use of Paro with cognitively-impaired older adults. This activity was simply interacting with the Paro robot on a participant’s lap and passing the robot around as users desired. Paro was brought to all but two of the sessions, acting as a more “active control-like” interaction due to its common use with older adults generally.

#### 6.2.1 Results

Residents of our partner SNF have experience with simple robotic toy dogs and cats, and so Paro acted as a familiar, if more advanced, intervention. Participants readily shared the robot with each other, passing it around and talking about it to each other and the research team. Specific participants were more likely to stay engaged with Paro than others. The majority of participants were content to watch those who were petting and playing with Paro, but they themselves declined interacting with the robot directly. The participants that were more likely to stay engaged with Paro were very engaged, with one non-verbal participant making joking gestures about stealing the robot back to their room.

### 6.3 Stretching with Stretch

**FIGURE 5:**
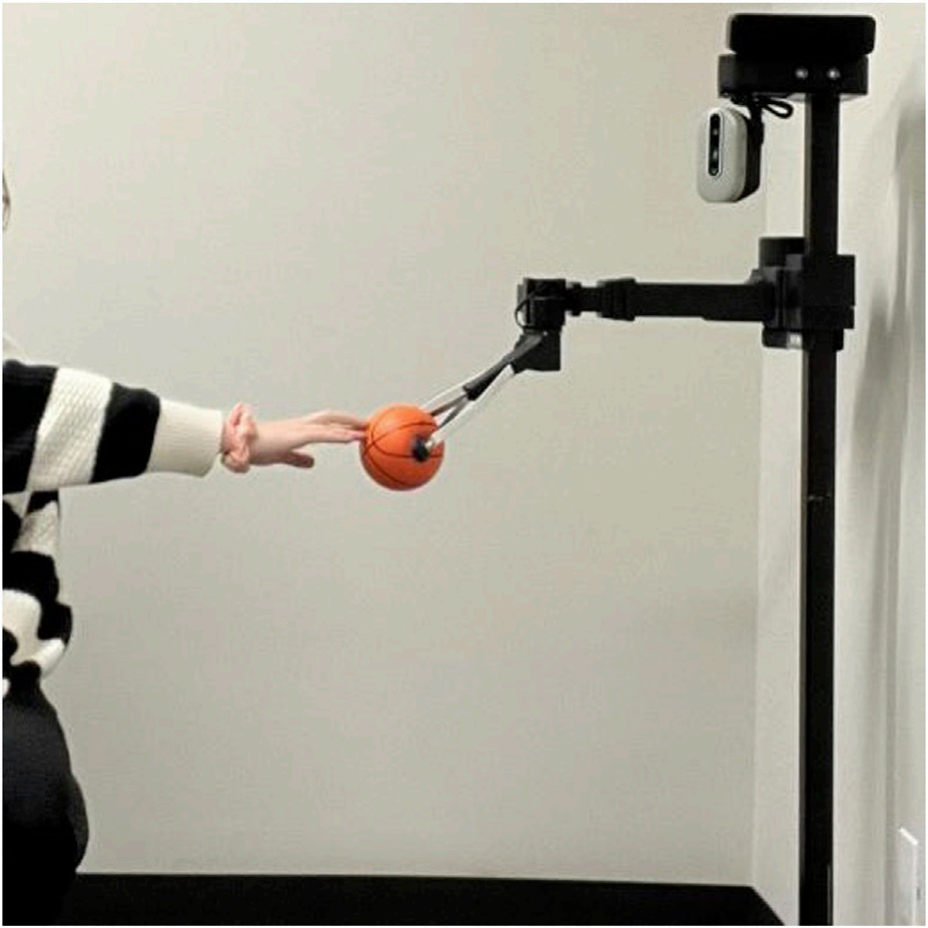
Stretch touch-based activity.

The Stretch robot was present for two sessions. The first simply allowed participants to control the system, while the second was a more structured stretching activity. Stretching with Stretch involved individual participants following stretching and reaching tasks with the Stretch mobile manipulator. This activity was based on the work presented in (Beatty et al., 2022) of performing physical activity tasks. Participants would reach with either a hand or foot to touch a ball that Stretch was holding, as the robot would move further away, raise the arm, or lower the arm to vary the stretching movement. An example of the reaching is presented in Figure 5.

#### 6.3.1 Results

Initial interactions with Stretch allowed participants to control the system, with one participant in particular using the system to playfully take items from the research team and place them in other locations. The second implementation was more controlled and focused on stretching and reaching motions with one participant. The participant played with the Stretch briefly but seemed uninterested in continuing to perform the actions.

### 6.4 Racing and battling with Cozmo

**FIGURE 6:**
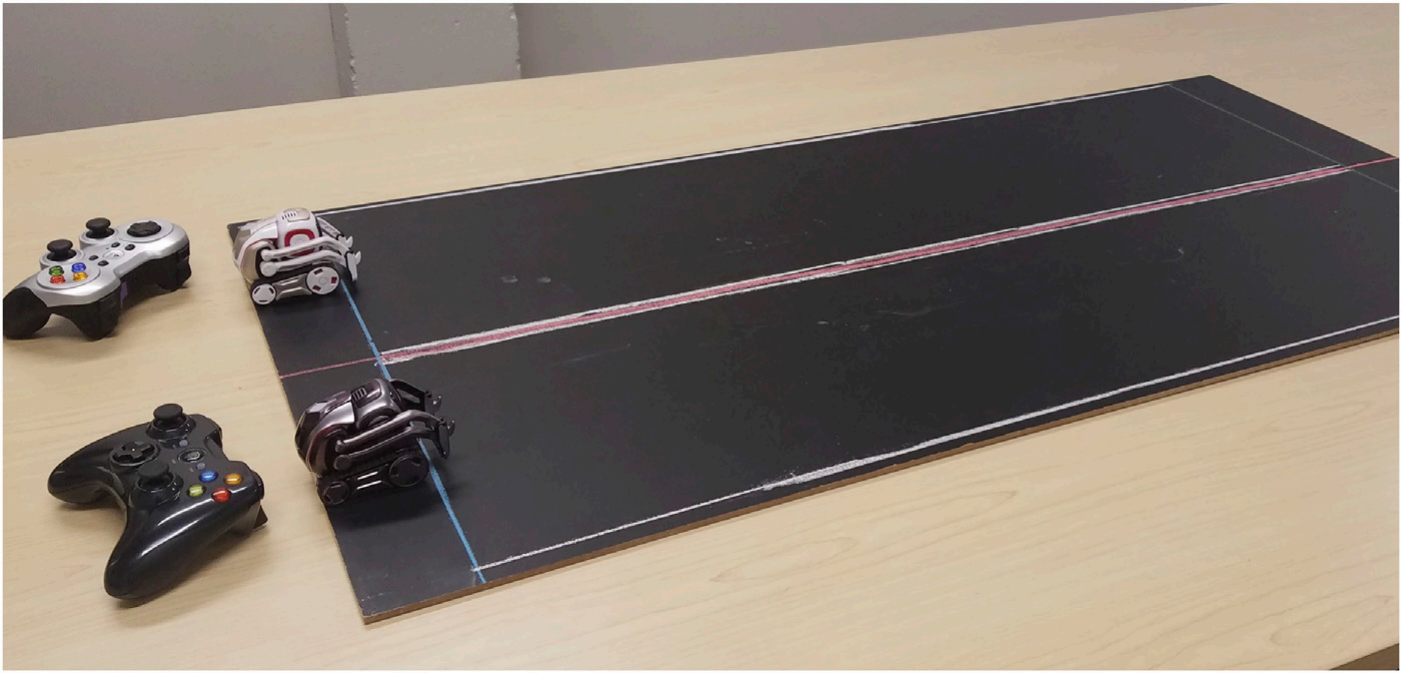
The layout of the chalkboard race track and two Cozmo systems with controllers as used in the racing activity.

Cozmo robots were included in two sessions. We initially brought a single Cozmo robot to observe resident ability to both see and notice the facial expressions, as well as how well they could use the game controller. After this, we implemented racing Cozmo, which led the residents to want to battle Cozmo. Racing involved pairs of participants racing the Cozmo robots down a small race track using video game controllers, as seen in Figure 6. Battling involved trying to push the other Cozmo out of a circular ring drawn on the same surface.

#### 6.4.1 Results

While many participants noticed when the Cozmo facial expression changed, they more frequently responded when the system would raise or lower either the head or front lift. Participants enjoyed providing orders and instructions to the system such as ordering it to “About Face” or “Salute” like a drill sergeant, or telling it to wave and nudge other individuals who were distracted or had just arrived. Additionally, while participants struggled to remember the layout of the controls, with the single robot they were able to recognize the problem and self-correct, or ask for a reminder of the controls without needing to be prompted by the research team. After confirming the visibility and control potential, we brought a second Cozmo and color-coded the systems (i.e., white Cozmo to a white controller, black Cozmo to a black controller) to help usability.

After several rounds of racing, residents began driving the Cozmos into each other, so we set up the impromptu battle arena, with enthusiastic reception. Two participants chose to control the Cozmos, and the remaining participants chose to watch the event. The two participants would occasionally get confused about which Cozmo was theirs when the Cozmos would switch sides of the arena.

### 6.5 Conversations with Misty

The Misty II robot was present was two sessions, first commentating on other activities, and then allowing participants to play with Misty’s text-to-speech functionality as part of a focus group on participants’ desired robot activities. Misty was initially used as a commentator during the Cozmo racing and battling, before being used for dedicated social interactions with and between participants.

#### 6.5.1 Results

Misty’s commentator role confused one participant in particular, who could not recognize that the audio was coming from Misty. However, they could clearly understand the audio, and so Misty was brought back for a more social session to talk about robots with the residents. Participants played with the Misty II control interface in a free-form way, and without prompting used it to communicate to each other. One participant, after having Misty make a particularly acerbic comment, told their non-verbal friend that this was how the individual imagined their non-verbal friend would talk.

### 6.6 Poker with PokerBot

**FIGURE 7:**
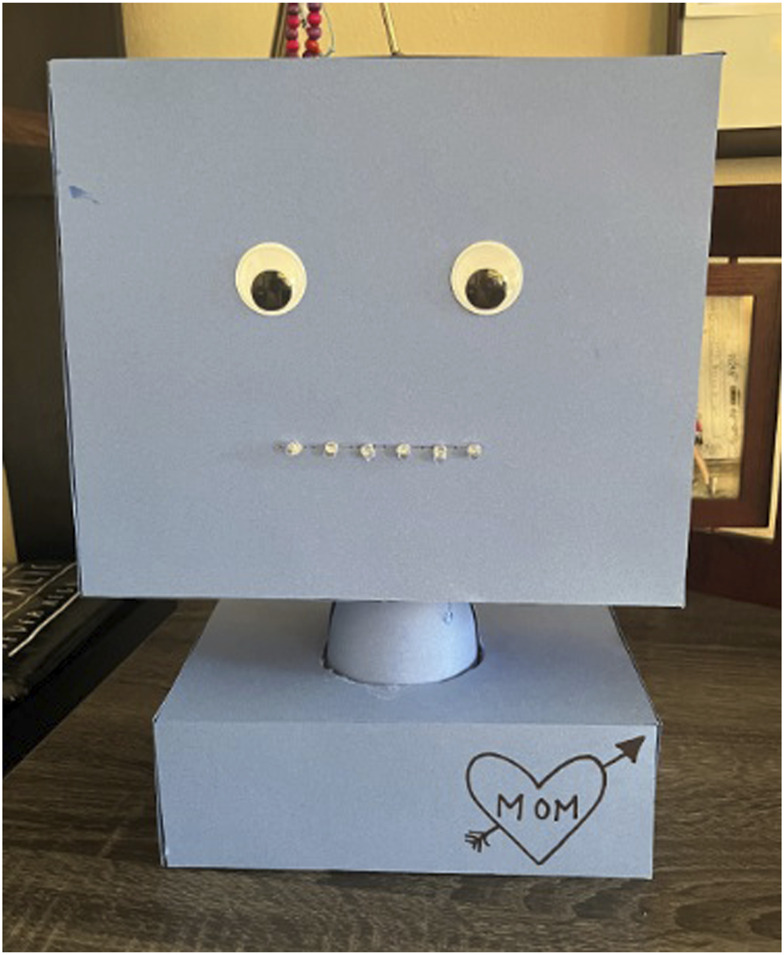
Final PokerBot design utilizing tabletop bust form factor and anthropomorphic qualities for the eyes and mouth.

Residents played poker with a custom robot head, dubbed PokerBot, as shown in Figure 7. PokerBot provided commentary and snarky repartee throughout the game. We used PokerBot for two sessions, during which one member of the study team dealt cards and another teleoperated the robot. The final tested design, as seen in Figure 7, was light blue, had googly eyes, and had green mouth LEDs that automatically blinked with the robot’s speech cadence. The blue and green color scheme was intentional; these are colors that are still visible in later stages of macular degeneration, which we learned was common among the residents. The robot had one degree of freedom for panning head rotation, along with a drawn on “tattoo” on its shoulder to represent the intended system character of being unrefined and impolite. The speech of the robot system was rendered using the Natural Reader text-to-speech application for iPhone at 1.06x speed, lower vocal range, and with a UK regional accent. This monotone, British, and male voice seemed to be fitting for the intended gruff personality of the system. Example quips from the system include “Saving up your energy to fold next round?” (in the case of a check) and “Did you guys hear about the cows that were out in the field, smokin’ weed, playin’ poker, and drinkin’ whiskey? The steaks were high!” (an ambient joke).

#### 6.6.1 Results

The residents enjoyed the PokerBot and found its attempts to antagonize them humorous. They laughed at the robot’s comments and interacted with each other to discuss the robot during the game. The residents occasionally struggled to hear what the robot was saying.

Despite the often crass culture of poker, the onlooking staff grew worried after the PokerBot made a particularly lewd comment during one session. While they were supportive of the initial idea, they voiced concern that a profane robot might come across as disrespectful to the residents. The residents did not seem to share this concern; they enjoyed the deployments and communicated interest in the system during a later session.

### 6.7 Mirroring with NAO

This activity included an initial session of testing with an Xbox 360 Kinect before the full game with the NAO. To add engagement to this test, we asked pairs of participants to watch the Kinect skeleton data of their partners on a screen and try to match their skeleton with that of the other participant.

We incorporated this pose matching with the NAO robot as a more involved game in which the NAO robot acted as a confederate in matching participant poses. This system was developed over multiple sessions, and was present for five of the sessions overall. The interaction concept involved a NAO robot mirroring participant movements and taking turns leading poses or following participant movements. As part of the pose leading, we developed multiple custom motion sequences which support the key movement types that were defined in Section 4.3, or were otherwise recognizable to participants, such as classic dance moves or movie references. Additionally, we developed custom audio cues for the range of interaction tasks, including speech to explain the activity, clarify whether a participant should be leading or following, prompt participants to perform the movement, react to participant movements and comments, prompt conversation, and interject jokes/other commentary. These audio and behavior cues were first tested with our gerontology collaborator and experts for appropriateness for residents, and several of the jokes were sourced from the residents during the sessions.

The system was the most developed of the systems tested in this work, and the underlying design process included the development of a full operator interface, kinematic conversions, custom behaviors for robot-led movements, and a variety of custom speech cues for engaging with participants. To help enhance related future research by other teams, we have open-sourced all aspects of this activity (i.e., behaviors, audio cues, NAO code, and operator interface) for broader usage (Preston et al., 2024).

#### 6.7.1 Results

During *testing* participants took turns leading and following, and regularly let other residents take a turn. We found that participants would individually tire of the activity, but be pulled back into interacting by other participants performing actions towards them on the monitor or verbally nudging them. In this way, we saw re-engagement prolong activity use.

With the NAO robot, more outgoing participants tended to enjoy leading the activity and watching the robot attempt to follow their movements, while quieter participants who wanted to interact were more likely to follow when the robot was leading. We also saw that participants could move very quickly when they were leading, but required much more slow and repetitive movements to successfully follow system poses. Similarly, we found that audio cues before the behaviors helped residents to be more responsive to each movement.

The Kinect did struggle to recognize participants that could not sit upright, or who kept their arms tight against their sides, which was not seen during the previous testing. Many of the SNF residents were wheelchair users, and struggled or were unable to readily sit upright to help the system recognize them.

### 6.8 Key highlights

Overall, our robot-based activities spanned different holistic support needs identified in our define phase. We found that activities that provided more control over the system, such as Cozmo racing or pose matching, tended to yield higher levels of focused attention, but did not necessarily result in more interaction between participants. Systems that provided the ability to interact with other participants, such as Misty’s text-to-speech or remote operation of Stretch/Cozmo were also positively received. Even if that was not the intent of the interaction, participants would find ways to use the systems to engage with each other whenever possible. Participants were more engaged in activities when there were opportunities to either directly interact with other participants or control the robot. The exception to this trend was Paro, which yielded either deep engagement or brief passing interest.

## 7 Discussion

In this work we examined the nuances, needs, and challenges present within SNFs, in addition to testing several robot-based activities within an SNF through an iterative design process with older adults and care providers in the loop. From this process we were able to define overarching challenges and needs within the space. This included barriers to resident activity engagement, such as a lack of perceived agency or unmet social and emotional needs, as well as types of activities or movement that will provide the broadest benefit to the overall health of residents. We incorporated these recommendations while prototyping robot-based activities for residents. We found through these prototype tests that residents enjoyed interacting with systems that put them in control of the robot in some way. However, we also saw that for many residents, either the robot or other residents were required to act as a social enabler to spur interaction with the system. The prototype tests allowed us to examine potential methods of improving overall engagement, as well as resident agency.

### 7.1 Design insights for the union of robotics and aging

Based on the results of our design process, we propose three most important overall insights to carry forward based on this work. These important insights for working with older adults in SNFs are, at a high level, 1) key barriers to wellness interventions, 2) prospective solutions for supporting wellness, and 3) design considerations which are important (and even well known) but often ignored. Although focused on the SNF setting in our work, several of these insights are more broadly applicable to older adults across the full spectrum of “old,” although they have more pronounced impacts for older adults. We note that these provocations include insights that recurrently appear in past related work; however, many have not been widely adopted in robotics research for older adults and thus merit reiterating.

#### 7.1.1 A digest of existing problems, to help yield more useful solutions

Motivating resident engagement with activities and each other can be challenging, for a wide range of reasons.

This difficulty arises in part because residents have reduced ability to do activities on their own, from brushing their teeth or getting out of bed, to going outside or having a cigarette. Many activities throughout the day (e.g., physical therapy, meals, or outings) are prompted or instigated by staff, which results in perceptions of both a lack of agency and loss of freedom. Results of this situation can manifest as unwillingness to participate in activities (e.g., PT), resistance to mobility aids and other assistive devices, and becoming a passive observer to staff attempts to instigate social engagement. Further, many “healthy” activities lack clear short-term rewards, and residents can experience difficulty remembering the activities and their purpose, which in turn can increase frustration. Feelings of isolation, loneliness, or a lack of adequate social interaction can also de-incentivize willingness to engage.

“Exercise” is a loaded word in the cultural context of our work, which leads to a number of different barriers to individual engagement. Namely, “exercise” is assumed to be boring or repetitive. The word “exercise” leads to assumptions about the difficulty level and required capabilities. Hearing “exercise” also results in people being afraid to be judged and pre-judging themselves as not good enough to start, or presuming that exercise is not “for them.”

High workload in SNFs result in high CNA turnover, and thus inconsistent training and prioritization of “secondary resident needs” such as social and physical activity, when compared to activities of daily living (e.g., eating). Even activities like bathing can be de-prioritized in cases such as staffing shortages or emergencies.

#### 7.1.2 Turning the tide with interaction and technology

Despite the broad challenges, past work and new observations from our efforts can potentially help to change the SNF wellness trends.

Group-based therapy and activities can improve self-efficacy, although outcomes are impacted by instructor mannerisms and overall group culture. Peer relationships are especially valuable for providing support and improving engagement in activities, and social companions can even lower barriers to joining or starting a new activity. We observed this in the way residents proactively assist each other during social events so that everyone can participate. Group- and peer-based interactions can lead to re-engagement or deeper engagement via verbal or physical peer-to-peer nudges that encourage participation.

Amidst the active research on robots for older adults and the common assumptions of reluctance for older adults to adopt new technologies, older adults in our partnering SNF are already interacting with robots such as low-cost companion dogs and cats, LEGO robots, Roombas, and assistive feeding systems, although the level of engagement or interest is resident-dependent.

In our test sessions, companion-type robots, including Paro, had either very high levels of continued engagement, or only brief acknowledgement. Similarly, for the systems with more active or passive engagement modes, such as leading or following with the pose-matching system, participants tended to prefer one mode more than the other during *ad hoc* interactions. Tactile interactions, in which participants either touched the robot or interacted with props, also appeared to be engaging.

When given control of the robots, residents used them to pester each other and physically interact. For example, they used Stretch to interact with the research team and each other by grasping and moving keys or poking one another. Residents liked ordering Cozmo around, for example, by verbally telling it commands or teleoperating it to go pester other residents. Participants used Misty’s text-to-speech capabilities to tease and talk to each other, including making crass comments and inside jokes.

#### 7.1.3 Design considerations for better robot interventions

Important goals of our proposed robotic interventions for SNF residents are to decrease feelings of helplessness, improve self-efficacy, and encourage resident social and physical activity. To improve engagement in residents with cognitive decline, it is important to provide reminders about how the activity or outcomes benefit the user. Additionally, for activities that participants might initially dismiss, providing a shift in framing or social support can improve overall interest and willingness to engage. For physical activity, key movements to focus on are cross-body reaching, weight shifting/waist movement, and foot reaching/lifting, which are important for resident balance and autonomy in daily activity. However, upper body work is more fatiguing, and quickly increases the heart rate, which may cause residents to fatigue more quickly. Keeping residents engaged, even when tired, is important, as even watching a robot or other people perform movements while imagining doing the same can improve later success.

Larger robots are easier to see, and though participants could see the small Cozmo robots, there were notable limitations of these smaller systems. Participants struggled to keep track of which Cozmo they controlled, even when there was color coding. Additionally, residents only noticed facial expressions on Cozmo if they were already focused on the robot. Otherwise, they only noticed larger movements of the robot. Screens displays require more mental processing to understand when trying to match body poses, such as during physical therapy exercises. Blue-green color schemes are more visible with later stage macular degeneration.

Participants struggled to fully hear and comprehend robot audio for multiple reasons. Hearing decline associated with age most strongly impacts higher-frequency audio (Fransen et al., 2003; Bunch, 1929), which impacts certain speech sounds and voice choices. This also means that robot audio needs to be clean and not “tinny,” and enunciation needs to be clear and precise. These issues can be partially addressed by increasing audio volume, but most robots use lower-quality speakers which tend to have poor audio quality (“tinniness”) as well as lower maximum volume. In addition to hearing decline, speed of auditory processing declines with age (Sharon et al., 1990; Tun et al., 2012), so the speed of any robot speech should be slowed and length of pauses increased to accommodate needed processing time. Further, systems need initial signalling that the robot is going to say something, so the first part of the speech is not missed due to processing delay. Residents struggled to tell where the audio voice was coming from with both Misty and NAO robots, potentially also due to a lack of clear visual cueing as to who was talking. Crass and snarky dialogue by the robots was enjoyed by participants, but of concern to staff due to the ease with which such dialogue could overstep social norms. The key design considerations that we present include visual, auditory, and behavioral characteristics of the robots themselves; for easy reference, a list of key guidelines is presented in Table 2.

**TABLE 2:** A synthesis of key design considerations for future robot designs in the SNF space.

Type	Guideline
Appearance	Larger size
Appearance	Contrasting colors or markings
Appearance	Blue-green color schemes
Audio	Lower-frequency cues
Audio	Limiting ‘sh’ type sounds
Audio	Clear audio and pronunciation
Audio	Slowing down audio cues
Behavior	Providing additional cues before audio information
Behavior	Longer pauses for user prompts
Behavior	Visual cues to help viewers localize audio source
Behavior	Slowing down movements that require user response

### 7.2 Limitations

While our efforts included a broad set of intervention activities and multiple *in situ* sessions, limitations of the work include being limited to a single SNF and a small pool of experts. We attempted to mitigate these limitations by incorporating repeated interactions with both residents and experts throughout the design process, in addition to expanding our group of experts as the design cycles necessitated. Further, while SNFs have many common traits, each facility has its own unique resident, staff, and operational attributes, which influences results in work like ours. Accordingly, in this work we needed to rely on our collaborators to understand what was most likely unique to our partnering SNF vs. what might be applicable to SNFs more broadly, as we worked with just one facility. Future work in this domain needs to include multiple SNFs to align with more generalized SNF needs and experiences. Additionally, while we saw residents engage and re-engage with our robotic systems, interactions were facilitated by a research team as part of the facility’s organized social activities. Responses to the interventions would likely be different without research assistant facilitators and scheduled events framing the activities. In our planned next research steps, we will seek to better understand more impromptu day-to-day interactions with the same types of robotic activities when they are installed as day-to-day elements of an SNF space for longer-term testing. These future steps will build on the design guidelines presented within the current paper.

## 8 Conclusion

Aging is a long process, and “younger” older adults have different needs and challenges than “older” older adults, who are more likely to live in an SNF than to “age in place.” SNFs, and their residents, have unique needs and challenges that have been under-explored relative to considerations for “younger” older adults, especially within assistive robotics research. Throughout the presented work, we focused on furthering the understanding of these needs and challenges, as well as exploring design prototypes for robot-based interventions to support SNF residents. The resulting design guidelines are intended to provide an important starting point for robot design within this space. Parts of our design thinking effort and our final proposed insights collect and hone common knowledge from outside of robotics for easy use and comprehension by roboticists. Further, our work fills in outcomes specific to our SNF, and possibly others like it, while also providing new knowledge about resident responses to a range of robot intervention prototypes. Generally, this work can support designing robots for and incorporating robots into SNFs in meaningful and beneficial ways.

## Data Availability

The datasets presented in this article are not readily available because the data discussed in this paper is not possible to release based on the study protocol; the video and text data that is the focus of the article cannot be shared ensuring anonymization. Requests to access the datasets should be directed to prestonr@oregonstate.edu.
